# How Milk Should Be Taken

**Published:** 1881-08

**Authors:** Dyce Duckworth


### How Milk Should be Taken.

Again, milk is a food that should not be taken in copious draughts like beer, or other fluids, which differ from it chemically. If we consider the use of milk in infancy, the physiological ingestion, that is, of it, we find that the sucking babe imbibes little by little the natural food provided for it. Each small mouthful is secured by effort, and slowly presented to the gastric mucous surface for the primal digestive stages. It is thus regularly and gradually reduced to curd, and the stomach is not oppressed with a lump of half-coagulated milk. The same principle should be regarded in the case of the adult. Milk should be slowly taken in mouthfuls, at short intervals, and thus it is rightly dealt with by the gastric juice. If milk be taken after other food, it is almost sure to burden the stomach, and to cause discomfort and prolonged indigestion, and this, for the obvious reason that there is insufficient digestive agency to dispose of it. And, the better the quality of the milk, the more severe the discomfort will be under these conditions.

Milk is insufficiently used in making simple puddings of such farinaceous foods as rice, tapioca, and sago. Distaste for these is engendered very often, I believe, because the milk is stinted in making them, or poor, skimmed milk is used. Abundance of new milk should be employed, and more milk, or cream, should be added when they are taken. In Scottish households this matter is well understood, and a distinct pudding-plate, like a small soup-plate, is used for this course. The dry messes commonly served as milky puddings in England are exactly fitted to create disgust for what should be a most excellent and delicious part of a wholesome dinner for both children and adults.—DR. DYCE DUCKWORTH, *in Popular Science Monthly for August.*

